# Medical student Internet usage: is the literature correct to call it addiction? An opinion piece

**DOI:** 10.3205/zma001351

**Published:** 2020-11-16

**Authors:** Ken Masters, Anne Herrmann-Werner

**Affiliations:** 1 Sultan Qaboos University, College of Medicine and Health Sciences, Medical Education and Informatics Department, Muscat, Oman; 2 University Hospital Tübingen, Medical Department VI/Psychosomatic Medicine and Psychotherapy, Tübingen, Germany

**Keywords:** medical students, internet addiction, communication

## Abstract

Over the past few years, there has been a sharp rise in the number of academic articles examining “Internet addiction” among medical students. This opinion piece views the Internet as a communication environment and a medical information tool within medical education. Within this context, the paper investigates the Internet Addiction Test (IAT) and criteria used in those articles, and questions their assumptions and conclusions. It then argues that what is often viewed as “addiction” may actually be dedication to work.

## Zusammenfassung

In den letzten Jahren gab es einen starken Anstieg der Anzahl akademischer Artikel, die die „Internetsucht“ von Studierenden der Medizin untersuchten. Der vorliegende Meinungsbeitrag betrachtet das Internet als eine Kommunikationsumgebung und ein medizinisches Informations-Tool innerhalb der ärztlichen Ausbildung. In diesem Kontext untersucht der Artikel den Internet Addiction Test (IAT) sowie die Kriterien, die in diesen Artikeln verwendet werden, und hinterfragt deren Annahmen und Schlussfolgerungen. Der Artikel argumentiert dann, dass das, was häufig als „Sucht“ angesehen wird, auch Hingabe an die Arbeit sein könnte.

## Introduction

### The Internet as a communication world

In 1969, greater and easier communication was at the heart of Leonard Kleinrock’s first experiments that gave birth to what was to become the Internet [1]. As the Internet developed and matured, the major technological influencers, such as Bill Gates and Tim Berners-Lee, emphasised the Internet’s vast communication potential, particularly in healthcare and education [2], [3]. Later, the advent of the iPhone and then other “smart-phones” saw the evolution of the “phone” into a powerful mobile computer, in which the “phone” is merely a computer application (“app”), among more than 2.5 million apps now available online from app stores [https://www.statista.com/statistics/276623/number-of-apps-available-in-leading-app-stores/].

Jakobson’s communication model [4], developed a few years before those first steps into the Internet, is as applicable today as it was 60 years ago, in spite of the fact that the Internet and mobile devices have introduced changes in the scope and nature of human communication, in which ubiquitous access to many-to-many communication is an expected part of life.

#### Internet immersion

The virtual world, however, can hold a fascination that is sometimes troubling. This is particularly so when the virtual world ceases to be an adjunct to the physical world, and the situation appears to be reversed. This reversal is already reflected in language changes: for some time now, “mail” has meant “e-mail”, and we describe paper letters as “snail-mail”; in online communication groups, “meeting” and “seeing” is taken to mean communication in the virtual world, and users speak of characters in the “meatworld” to refer to people in the physical world.

#### Online addiction

More troubling, however, is that the amount of time spent online demonstrates characteristics of an addiction. There are so many articles on the topic of Internet addiction that a full systematic review on the topic, beyond the scope of this paper, would be required to give a proper sense of the number and scope of the discussion, but it is safe to say that the discussion appears to have long moved past whether or not one can speak of such addiction, but rather the extent to which the addiction exists. In fact, many papers no longer speak of Internet addiction in the general sense, but rather focus on social media, or particular instances of social media, such as Facebook.

For example, a 2014 literature review on Facebook addiction [5] found 24 studies on the topic: this was when Facebook was barely 10 years old.

Medical students’ addiction to virtual activities has also increasingly been the subject of detailed studies. The authors of this paper conducted a brief PubMed search with the search phrases “medical students” and “Internet addiction” published since September 2009 (covering a 10-year period) and received 42 results, almost all of them published in 2018 or 2019.

For their measurement tools and criteria, these papers typically use variations of the Internet Addiction Test (IAT) [6], [7], [8], and then report on statistics in broad scales of Internet usage [7], [8], [9], or more specific details, such as “The majority (82.3%) of participants reported at least frequently staying online longer than intended” “…and that they lost sleep because of late-night Internet use (70.8%)” and “[s]tudy respondents also reported health problems including headaches, backache, weight gain, neck pain and other psychological problems as a result of Internet use” [6].

#### Online activity

There does appear to be great reason for concern over these statistics, the notion that medical students are addicted to the Internet, and that they are being so heavily negatively impacted by this addiction.

But do these papers present a balanced and fair view of the situation? In this opinion piece, we aim to deconstruct the general narrative in order to shed more light on the topic. We begin by describing the history and characteristics of the IAT, and then examine the nature of the Internet as a complex environment and modern-day library, and then reflect again on the IAT in light of that examination. We then assess the literature sources in detail, questioning their assumptions about Internet usage, and their confusion of correlation with causation. We then discuss the shift in communication approaches brought about by the Internet, and end with a brief view of possible future investigations, so that we can more fairly assess the extent to which there is reason for concern about Internet addiction among the world’s medical students.

## Internet addiction

### The Internet Addiction Test

We begin by noting that there is not one single Internet Addiction Test (IAT). The first known attempt to construct such a tool was by Kimberly S Young in 1998 [10], who used the *Diagnostic and Statistical Manual of Mental Disorders IV* (DSM-IV), Pathological Gambling questionnaire as a guide, and developed eight questions. Since then, based upon these and additional criteria, other researchers have constructed question lists of roughly 20 questions, usually claiming to be based upon DSM-IV and DSM-5.

Extracted from various references, such as [6], here are of some of the questions in the IAT to which one would respond with “Occasionally”, “Frequently”, “Often” or “Always”.

#### How often do you:

- stay online longer than intended?
- neglect household chores to spend more time online?
- form new relationships online with fellow Internet users?
- [have] others complain about the amount of time you spend online?
- snap, yell or act annoyed if someone interrupts you while you are online?
- lose sleep due to late-night Internet use?
- find yourself saying “just a few more minutes” when online?
- choose to spend more time online than going out with others?

At first glance, one could easily assume that the validity of these questions contributes to the image of an addiction if the respondent answers many of the questions with "often" or "always". We need to remember, however, that the word “addiction” is a clinical diagnosis (incorporating many other facets, such as duration), and is not to be used lightly. In addition, Internet Addiction Disorder is not currently recognised as a disorder in the *Diagnostic and Statistical Manual of Mental Disorders, Fifth Edition *(DSM-5) [11] or its 2018 Supplement [12], in spite of some belief that it is [6]. For that reason, we need to examine the Internet in a little more detail, and review these question in light of that examination.

## The nature of the Internet

### As a complex work-related environment

The Internet is a network of highly disparate objects. When one speaks of online or the Internet, one does not speak of a simple item, but rather a vast sphere of more than 1.7 billion websites [https://www.internetlivestats.com/total-number-of-websites/] and at least 4.5 billion people [https://www.internetworldstats.com/stats.htm].

Among the most relevant aspect of the Internet to this paper is the Internet as a place of work, particularly for medical students. Medical education is rich with research on how the Internet and aspects of it (including methods of e-learning, m-leaning and artefacts such as specific social media) should, can and are to be used by medical students and professionals [13], [14], [15], [16]. In medical education, the Internet is the meeting place of humans and the information they require for their work. In Engeström’s *Activity Theory*, it is squarely positioned as a work tool or instrument or mediating artefact [13], [17]. It is, in effect, what we used to call a library.

#### As a library

Those of us who first attended university before 2000 may recall the hours of studying we performed in the library. So, we look again at those “addiction” questions, but this time, replace “online” or “Internet” with “studying” or “library”. It would be a useful exercise for any readers who obtained their degree before the year 2000 to answer these questions with “Occasionally”, “Frequently”, “Often” or “Always”.

#### How often did you:

- stay [in the library] longer than intended?
- neglect household chores to spend more time [studying or in the library]?
- form new relationships with fellow [library] users?
- [have] others complain about the amount of time you spend [studying or in the library]?
- snap, yell or act annoyed if someone interrupts you while you are [studying]?
- lose sleep due to late-night [studying or library] use?
- find yourself saying “just a few more minutes” when [studying or in the library]?
- choose to spend more time [studying or in the library] than going out with others?

In addition, if any 20^th^ century student can honestly say that they did not suffer from “health problems including headaches, backache, weight gain [or unintended weight loss], neck pain and other psychological problems” [6] as a result of studying, especially in those, oh, so uncomfortable university library chairs, then we would love to know how they obtained their degree in the first place. And that does not even mention skipped meals, forgotten doctors’ appointments, forgotten birthdays – it is common knowledge that obtaining a PhD and family relationships….well perhaps the less said about that, the better.

Based on this, if these questions from the IAT had been put to you, you might very well have found yourself in line to being diagnosed as addicted to your studies and the library. Either that, or you were a conscientious student motivated by achieving good grades, or perhaps one who understood that one day, you would have patients’ lives in your hands, and that you needed to utilise your studying time to the best of your ability.

## The sources

Above, we cited a few sources that showed the impact of extended Internet usage. If we look at those sources in a little more detail, we notice a few trends:

### No indication of work

Many of the cited sources give no indication of any student academic work being done on the Internet, almost as if they are unaware of this aspect of the Internet. Where researchers do mention academic work, it is almost in passing and then ignored [7]. In fact, when the addiction is measured, the problem is clearly framed in terms of Internet vs. work, and the actual activities are not examined in any detail [6], [7], [8], [18]; it is as if Internet activities and student academic work are mutually exclusive.

Why this is so is not entirely clear. Either the authors decided that the specific activities were not important enough to investigate, or, if they did investigate it, they have withheld that information. This is in spite of the fact that some of the authors acknowledge in their introduction that the Internet serves an educational purpose [7], [9], [18], and others (e.g. [6]) have even have read and cited a paper [19] that specifically demonstrates that, when one takes into account that much of the time spent by students on the Internet is work-related, then “addiction” figures drop by approximately 50%.

Ignoring the contribution of the Internet to work was already implicit in Young’s original eight criteria, where item 6 asked “Have you jeopardized or risked the loss of a significant relationship, educational or career opportunity because of the Internet?”. The implication here is that one’s job or career or education was in opposition to the Internet rather than possibly reliant upon it. In 1998, this attitude was, perhaps, understandable: in spite of Bill Gates’ vision of the Internet [2], many experts had, as late as 1995, still seen the serious and business prospects of the Internet as “Baloney” [20]. After 2010, however, one is at a loss to explain how a researcher at a modern university, reliant for every aspect of their work on the Internet, could not take into account that much of Internet “addictive” behaviour could be strongly motivated by work and study pressure.

#### Confusing correlation with causation

It is also telling that those studies that find a correlation between Internet “addiction” and poor academic performance tend to ascribe a one-way causal effect: Internet usage leads to poor performance [7], [18]. The papers do not raise the possibility of a reverse causality: students with lower performance abilities are trying to improve their understanding (and grades) by using the Internet for academic activities such as reading materials and watching instructional videos. The researchers do not address the obvious contradiction between their causal inference and other studies that show the educational benefits of the Internet. It is almost as if the Khan Academy [https://www.khanacademy.org/] does not exist. It is really unlikely that the more than 3.5 million people who have watched the Academy video on blood flowing through the heart [https://www.youtube.com/watch?v=7XaftdE_h60] and the more than 1 million people who have watched a video on linear regression [https://www.youtube.com/watch?v=ZkjP5RJLQF4] have done so because they were “addicted” to the Internet, and not struggling students attempting to understand complex topics.

This attitude is reflected in other, more subtle ways. For example, Grover et al. found an association between Internet “addiction” and burnout and stress, and gave one unsubstantiated suggested conclusion that the students have burnout “because they are not able to spend the desired time for using the Internet” [8]. It is a pity that the authors did not consider another possibility: working harder leads to burnout, and so using the Internet to work more could be associated with, and cause, burnout.

Similarly, Grover et al. find a correlation between “addiction” and the students’ observing “that the seniors do not show empathy towards patients” [8]. Given that so much research indicates a reduction in patient empathy as students progress through their medical degrees, one would expect seniors to show relatively less patient empathy, and so it is a good thing that it is being observed. An interesting question to research would be “why are the Internet “addicted” students noticing this more than other students?”

#### Lack of reflection on their research

Somewhat ironically, these researchers’ attitudes towards the Internet, as something harming work, exist while those researchers themselves would have spent many hours on the Internet researching their topic and preparing, writing and submitting their manuscripts. Perhaps they might have reflected on the amount of time they spent on the Internet, and wondered if this time indicated Internet “addiction” or simply dedication to one’s task – the same level of dedication that we would want to see in medical students.

As medical educators, we have frequently seen students’ engaging with their mobile devices and laptops in the classroom and on the wards. It would be naïve to assume that they are all working; but it would be equally at fault to ignore the possibility that many of them are engaged in educational activities, such as pre-reading materials, looking up explanatory information (including definitions, translations, and simpler synonyms), or querying misunderstandings with other students in the class.

## A shift in communication

### Communication, but not with those physically present

This paper began with a description of the early Internet development as a communication tool. This ability to encourage communication lies at the heart of the Internet’s value, and in many cases, the great amount of device and social media usage is simply communication [5]. Perhaps what is distracting is that the people using the device to communicate are not communicating with others who are physically present in the “meatworld.”

While the person on the device may be viewed as selfish, one could argue that the others who are physically present are selfish – making an unreasonable demand to continuously be the focus of the communication. Perhaps we rate too highly one’s physical presence, and, for some reason, believe that our being physically present has importance that overrides the importance of the people with whom the device holder is communicating online. There is surely nothing to justify our perceived importance, other than social convention. Social conventions, however, change. Why should our current set of conventions (apparently rapidly fading), have any special prominence?

There may be legitimate concerns about how communication with wider groups may weaken relationships with immediate groups, especially family members; this is then surely best addressed though strengthening of filial relationships, and how we use communication to develop and maintain them. This view is in line with the Guidelines offered by the Royal College of Paediatrics and Child Health [21] who, while indicating that too much screen time by children can be harmful, offer guidelines focusing on family activities, including accessing materials together, much in the way that our parents were advised to effectively use television.

#### Appreciation for communication, not one’s social or professional status

There is also an important communication aspect to the Internet regarding a fairer appreciation of one’s abilities based on contribution and no other irrelevant characteristics. Although there are varying degrees of online status, in many online situations, communication and contribution is anonymous. This has the unique advantage that people are valued because of their contributions to the communication or tasks at hand, and not by any real-world status. It does not matter if you are a teenager or a professor: what matters is your contribution. Perhaps a prime example is given by Cory Doctorow, who recalls the online programming team that was developing the early computer protocols for RSS and XML, and speaks of one programmer, Aaron Swartz, who was 

*combatative, but very smart, and who had lots of good ideas, and he didn’t ever come to the face-to-face meetings, and they [the other programmers] said,“When are you going to come out to one of these face-to-face meetings?” and he [Aaron] said,“You know, I don’t think my mom would let me, you know, I just turned fourteen.” And so, their first reaction was, “This person, this colleague we’ve been working with all year, was thirteen years old while we were working with him, and he’s only fourteen now.” And their second reaction was “…we really want to meet him.” *[22]

We are not all Aaron Swartz, but when people’s communication is valued because of its contribution to the conversation, rather than because of their age, gender or other characteristics, then it is little wonder that they will turn to the virtual world rather than the “meatworld”.

## Future work: identify the activities

It is telling that, while “Internet Addiction Disorder” is not listed in the *Diagnostic and Statistical Manual of Mental Disorders, Fifth Edition* (DSM-5) [11], nor the ICD11 codes, DSM-5 lists “Internet Gaming Disorder”, and ICD11 lists “Gaming disorder, predominantly online” (6C51.0). This gives us a clue as to where to go from here. Recognising that Internet activities, and even social media usage, are far too diverse to be lumped under a single heading, it would be more useful if researchers identified specific activities and then examined those. It is also possible that there is an “addiction” but not to the Internet; given the time that medical students spend in this vast library, it possible that this is an addiction to work.

## Conclusion

The Internet, as envisaged by its earliest creators, is a vast network facilitating human communication. For medical students, a crucial aspect of this communication is the ability to retrieve information, and to contribute their own insights. In spite of this, research into medical students’ Internet usage has led to concerns about Internet addiction. This opinion piece has argued that these concerns have frequently resulted from researcher error, in which the researchers use a flawed test, ignore the Internet’s value as an electronic library and a collaborative learning and working environment, confuse correlation with one-way causation, ignore their own and others’ opposing evidence, and the nature of Internet communication.

While there may be an addictive quality to medical students’ Internet usage, we know that they (and most other students) are using the Internet as a vast wealth of knowledge and as a communication phenomenon, and, until we examine their activities and reasons behind those activities in detail, we should be very reluctant to classify their Internet usage as “addiction.”

## Competing interests

The authors declare that they have no competing interests. 

## References

[1] Kleinrock L, Norman JM (2005). UCLA to be First Station in Nationwide Computer Network. From Gutenberg to the Internet: A Sourcebook on the History of Information Technology.

[2] Gates B (1995). The Road Ahead.

[3] Berners-Lee T, Hendler J, Lassila O (2001). The Semantic Web. Sci Am.

[4] Jakobson R (1960). Closing Statement: Linguistics and Poetics. Style in Language.

[5] Ryan T, Chester A, Reece J, Xenos S (2014). The uses and abuses of Facebook: a review of Facebook addiction. J Behav Addict.

[6] Taha MH, Shehzad K, Alamro AS, Wadi M (2019). Internet use and addiction among medical students in Qassim University, Saudi Arabia. Sultan Qaboos Universiity Med J.

[7] Asokan AG, Varghese VA, Rajeev A (2019). Internet addiction among medical students and its impact on academic performance: an Indian study. J Med Sci Clin Res.

[8] Grover S, Sahoo S, Bhalla A, Avasthi A (2019). Problematic internet use and its correlates among resident doctors of a tertiary care hospital of North India: A cross-sectional study. Asian J Psychiatr.

[9] Javaeed A, Zafar MB, Iqbal M, Ghauri SK (2019). Correlation between Internet addiction, depression, anxiety and stress among undergraduate medical students in Azad Kashmir. Pakistan J Med Sci.

[10] Young KS (1998). Internet Addiction: the emergence of a new clinical disorder. Cyber Psychol Behav.

[11] American Psychiatric Association (2013). Diagnostic and Statistical Manual of Mental Disorders.

[12] American Psychiatric Association (2018). DSM-5 Update October 2018: Supplement to Diagnostic and Statistical Manual of Mental Disorders, Fifth Edition.

[13] Masters K, Ellaway RH, Topps D, Archibald D, Hogue RJ (2016). Mobile technologies in medical education: AMEE Guide No. 105. Med Teach.

[14] Coleman E, O'Connor E (2019). The role of WhatsApp® in medical education; a scoping review and instructional design model. BMC Med Educ.

[15] O'Sullivan C, Janssens S, Warhurst K (2019). BOGGLE Your Brain: An Online Forum for Obstetrics and Gynaecology Graduate Medical Education in a Tertiary Maternity Hospital. MedEdPublish.

[16] Wanner GK, Phillips AW, Papanagnou D (2019). Assessing the use of social media in physician assistant education. Int J Med Educ.

[17] Engeström Y (2001). Expansive Learning at work: Toward an activity theoretical reconceptualization. J Educ Work.

[18] Akhter N (2013). Relationship between Internet addiction and academic performance among university undergraduates. Educ Res Rev.

[19] Masters K (2015). Social networking addiction among health sciences students in Oman. Sultan Qaboos Univ Med J.

[20] Stoll C (1995). Why the Web won't be Nirvana. Newsweek. Newsweek.

[21] RCPCH (2019). The health impacts of screen time: a guide for clinicians and parents.

[22] Knappenberger B (2014). The Internet's Own Boy: The story of Aaron Swartz.

